# The effect of capsaicin on expression patterns of CGRP in trigeminal ganglion and trigeminal nucleus caudalis following experimental tooth movement in rats

**DOI:** 10.1590/1678-775720160150

**Published:** 2016

**Authors:** Yang ZHOU, Hu LONG, Niansong YE, Lina LIAO, Xin YANG, Fan JIAN, Yan WANG, Wenli LAI

**Affiliations:** Sichuan University, West China Hospital of Stomatology, Department of Orthodontics, State Key Laboratory of Oral Diseases, Chengdu, China.

**Keywords:** Capsaicin, Calcitonin gene-related peptide receptors, Tooth movement, Trigeminal ganglion, Trigeminal caudal nucleus

## Abstract

**Objectives:**

The aim of this study was to explore the effect of capsaicin on expression patterns of calcitonin gene-related peptide (CGRP) in the trigeminal ganglion (TG) and trigeminal subnucleus caudalis (Vc) following experimental tooth movement.

**Material and Methods:**

Male Sprague-Dawley rats were used in this study and divided into small-dose capsaicin+force group, large-dose capsaicin+force group, saline+force group, and no force group. Closed coil springs were used to mimic orthodontic forces in all groups except for the no force group, in which springs were inactivated. Capsaicin and saline were injected into periodontal tissues. Rats were euthanized at 0 h, 12 h, 1 d, 3 d, 5 d, and 7 d following experimental tooth movement. Then, TG and Vc were obtained for immunohistochemical staining and western blotting against CGRP.

**Results:**

Immunohistochemical results indicated that CGRP positive neurons were located in the TG, and CGRP immunoreactive fibers were distributed in the Vc. Immunohistochemical semiquantitative analysis and western blotting analysis demonstrated that CGRP expression levels both in TG and Vc were elevated at 12 h, 1 d, 3 d, 5 d, and 7 d in the saline + force group. However, both small-dose and large-dose capsaicin could decrease CGRP expression in TG and Vc at 1 d and 3 d following experimental tooth movement, as compared with the saline + force group.

**Conclusions:**

These results suggest that capsaicin could regulate CGRP expression in TG and Vc following experimental tooth movement in rats.

## INTRODUCTION

Orthodontic pain induced by tooth movement is widely considered as a negative consequence of orthodontic treatments^19^. Although the perception of pain is a subjective experience and might vary in individuals, almost all patients complain the discomfort during orthodontic treatment^3^. Therefore, how to alleviate orthodontic pain and elucidate its underlying mechanisms clearly represent one of the major concerns for both patients and orthodontists. Orofacial pain signals induced by tooth movement are received by peripheral nociceptors, transmitted to the first-order neurons in the trigeminal ganglion (TG), conveyed to the trigeminal subnucleus caudalis (Vc) located in the caudal part of medulla oblongata, then relayed to the third-order neurons in the thalamus, and finally perceived by the cortex^19^. Well-grounded experimental observations have documented that both TG and Vc play a crucial role in the transmission of orthodontic pain^16^ ^,^ ^30^.

It is well known that the perception of orthodontic pain is probably due to an inflammatory reaction localized in periodontal tissues that involves the release of various inflammatory mediators^28^. In particular, calcitonin gene-related peptide (CGRP), a representative neuropeptide, is evidenced to participate in the initiation and maintenance of inflammatory pain^2^. Moreover, it has been demonstrated that CGRP participates in orthodontic pain following experimental tooth movement^18^. Capsaicin, the main pungent ingredient in hot chili peppers, could elicit heat sensation and stimulate the release of sensory neuropeptide through selectively binding to transient receptor potential vanilloid 1 (TRPV1, a non-selective cation channel)^6^. CGRP could be synthesized and released from a subset of capsaicin-sensitive primary afferent neurons in the TG^12^. However, the relationship between capsaicin and CGRP release following tooth movement is still poorly understood. Although previous studies have indicated that capsaicin-sensitized TRPV1 could evoke the CGRP release from peripheral nerve axons^13^, the effect of capsaicin on CGRP expressions in TG and Vc following experimental tooth movement remains largely unknown. Furthermore, administration of capsaicin does not affect the rate of orthodontic tooth movement in rats^9^.

Therefore, the purpose of the present study was to investigate the effect of capsaicin on expression patterns of CGRP in TG and Vc following experimental tooth movement in rats and to explore its underlying mechanisms.

## MATERIAL AND METHODS

All experimental procedures were conducted according to the National Institutes of Health Guide for the Care and Use of Laboratory Animals, and were approved by the Ethical Committee of West China School of Stomatology, Sichuan University and State Key Laboratory of Oral Diseases. Efforts were made to minimize both the number of animals and their discomfort.

### Animals

Male Sprague-Dawley rats (age: 8 weeks; weight: 200-250 g) were used in this study. These rats were provided by the Animal Experimental Center, Sichuan University, Chengdu, China. They were housed in a temperature-regulated room at 25±2°C with standard rat chow and water *ad libitum*, and maintained on a 12/12 day-night cycle in a quiet environment for at least five days prior the experiment.

### Experiment design

In order to examine the effects of capsaicin on CGRP expression following experimental tooth movement, rats were randomly divided into small-dose capsaicin+force group, large-dose capsaicin+force group, saline+force group, and no force group. Fixed and stainless steel closed-coil springs were ligated between left upper first molars and the ipsilateral upper incisors to delivery a mesial force of 40 g as previously described^23^ in the force groups while springs inactivated in the no force group (0 g). Rats were fed with soft food after force application. They were euthanized through cervical dislocation following general anesthesia at 0 h, 12 h, 1 d, 3 d, 5 d, and 7 d (six rats at 0 h, 12 h, 1 d, 3 d, 5 d, and 7 d in each group). In particular, in all groups, the rats euthanized at 0 h without any intervention were taken as the baseline control for each group.

The rats of the small-dose capsaicin+force group were injected with 20 μL of capsaicin solution (3×10^-2^ mol/L; Sigma-Aldrich, St. Louis, MO, USA)^15^ into periodontal tissues around upper left molars at four different sites (5 μL/site), while rats of the large-dose+force group were injected with 100 μL (25 μL/site). The dosage of capsaicin used in this study has been shown to be effective in a previous study^31^. Moreover, 0.9% saline (20 or 100 μL) was used in the saline+force group. Periodontal injections were performed 30 min before force application.

### Tissue sample preparations

Following cervical dislocation, the maxillary portion of TG at the ipsilateral side of force application and the caudal part of medulla oblongata were obtained for immunohistochemistry and western blot.

### Immunohistochemistry

The expressions of CGRP in TG and Vc were detected by immunohistochemistry at six time points (0 h, 12 h, 1 d, 3 d, 5 d, and 7 d) after experimental tooth movement in rats (n=6 *per* group). Tissue samples were fixed with 4% paraformaldehyde, washed in phosphate-buffer saline (PBS) three times, and then embedded in paraffin. Serial tissue sections were cut at a thickness of 10 μm and then deparaffinized. After antigen retrieval (microwave, citrate acid buffer), the sections were washed in PBS, blocked with goat serum, and incubated with a specific rabbit anti-CGRP antibody (1:200; ab47027; Abcam, Cambridge, MA, USA) at 37°C for 45 min. After that, sections were rinsed with PBS for 10 min and incubated with EnVision^TM^ (K500711; DAKO, Carpinteria, CA, USA) at 37°C for 45 min. Following washing with PBS for 10 min, they were visualized with 3,3’-diaminobenzidine (DAB) and washed with distilled water. After counterstained with hematoxylin and dehydrated with ethanol, immunostained sections were mounted on coverslips. All tissues followed the same immunohistochemical procedures to minimize the variability in laboratory.

Visualization of immunoreactive tissues was obtained through a light microscope (Axio Imager 2, Carl Zeiss, Oberkochen, German). The positive CGRP expression was defined as yellow-brown-stained cytoplasm for TG and yellow-brown stained reticular formation for Vc. For each rat, integrated optical density (IOD/Area) for TG and Vc were calculated (Image-Pro Plus 6.0, Media Cybernetics, Rockville, MD, USA) in each of five randomly consecutive fields in each of three slides, and the mean of these 15 fields was employed as CGRP expression level.

### Western blotting

The CGRP protein expression in TG and Vc were studied by western blotting at six time points after experimental tooth movement in rats (n=6 *per* group). Following mechanical grinding, tissue samples were disintegrated with RIPA lysis buffer on ice for 30 min and centrifuged at 4°C Then, the supernatants were extracted and stored at -70°C. After determining the total protein concentration, samples were separated by 15% SDS-PAGE and transferred to polyvinylidene fluoride (PVDF) membranes. They were washed four times and blocked with 5% defatted milk powder for 2 h, then incubated with a specific rabbit anti-CGRP antibody (1:200; ab47027, Abcam, Cambridge, MA, USA). The membranes were then washed in PBS and incubated with secondary antibody, Dylight 680 conjugated goat anti-rabbit IgG (1:10000, KPL). β-actin was used as an internal control protein. Band intensity was computer analyzed by a densitometer (Quantity One, Bio-Rad, Hercules, CA, USA). Each band was analyzed repeatedly three times, and the mean ratio of CGRP/β-actin was employed as the CGRP expression level.

### Statistical analyses

Data were expressed as mean±standard error (SEM). One-way analysis of variance (ANOVA) (Tukey *post hoc* test) was applied to analyze differences of CGRP expression levels among different time points in each group and to compare the differences of CGRP expression levels among the four groups at each time point. P values less than 0.05 were considered to be statistically significant. All the statistical analyses were performed in SPSS 19.0 (SPSS, Chicago, IL, USA) and GraphPad Prism 6.0 (GraphPad software, San Diego, CA, USA)

## RESULTS

### The effects of capsaicin on CGRP expression in the TG following experimental tooth movement

**Figure 1:**
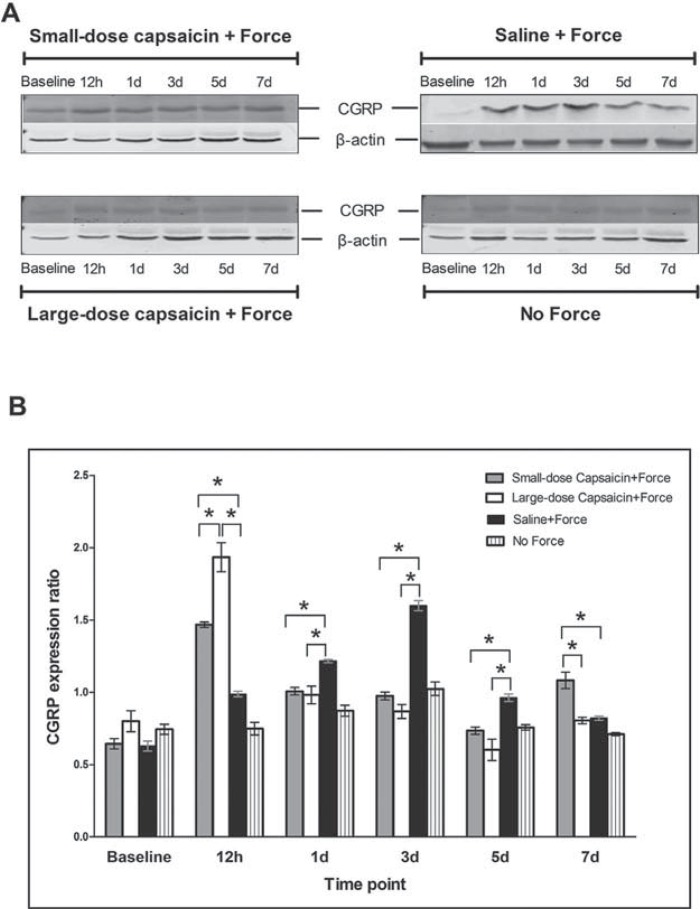
CGRP expression levels in trigeminal ganglion (TG) following experimental tooth movement detected by western blotting. A: CGRP bands in TG at different time points in the four groups; B: The histogram showed the expression ratio of CGRP protein levels normalized to that of β-actin in the same sample. * indicates p<0.05

As displayed in Figure 1A, bands of CGRP were shown near to 38 kDa, and that of β-actin near to 42 kDa.

For the saline+force group, when compared with the baseline level (0.628±0.041), as shown in Figure 1B, experimental tooth movement induced an up-regulation of CGRP expression in the saline+force group at 12 h (0.988±0.019, p<0.001) and 1 d (1.216±0.011, p<0.001), peaked at 3 d (1.599±0.034, p<0.001), then decreased gradually at 5 d (0.962±0.026, p<0.001) and 7 d (0.822±0.015, p=0.002<0.05). For the no force group, when compared with the baseline level (0.745±0.035), the CGRP expression levels were only increased at 3 d (1.024±0.046, p=0.001<0.05).

Moreover, CGRP expression levels in the small-dose capsaicin+force group were significantly higher than in the saline+force group at 12 h (1.468±0.019 vs. 0.988±0.019, p<0.0001), then greatly decreased and became significantly lower than in the saline+force group at 1 d (1.007±0.026 vs. 1.216±0.011, p<0.05), 3 d (0.974±0.027 vs. 1.599±0.034, p<0.0001), and 5 d (0.734±0.025 vs. 0.962±0.026, p<0.05), whereas it increased again ( 1.082±0.056 vs. 0.822±0.015, p=0.0001) at 7 d. Similarly, CGRP expression levels in the large-dose capsaicin group were also significantly higher than in the saline+force group at 12 h (1.934±0.101 vs. 0.988±0.019, p<0.0001), then decreased and were significantly lower than in the saline+force group at 1 d (0.982±0.061 vs. 1.216±0.011, p<0.05), 3 d (0.868±0.048 vs. 1.599±0.034, p<0.0001), and 5 d (0.602±0.074 vs. 0.962±0.026, p<0.0001); however, the CGRP expression levels finally became similar between the two groups (0.805±0.022 vs. 0.822±0.015, p=0.9797>0.05) at 7 d.

Interestingly, regarding the comparison between the two groups with different capsaicin dosages, we found that CGRP expression levels were significantly higher in the large-dose capsaicin+force group than in the small-dose capsaicin+force group at 12 h (p<0.05). Conversely, following the similar CGRP expression levels between these two groups at 1 d, 3 d, and 5 d (all p>0.05), CGRP expression levels were significantly higher in the small-dose capsaicin+force group than in the large-dose capsaicin+force group (p<0.05).

**Figure 2:**
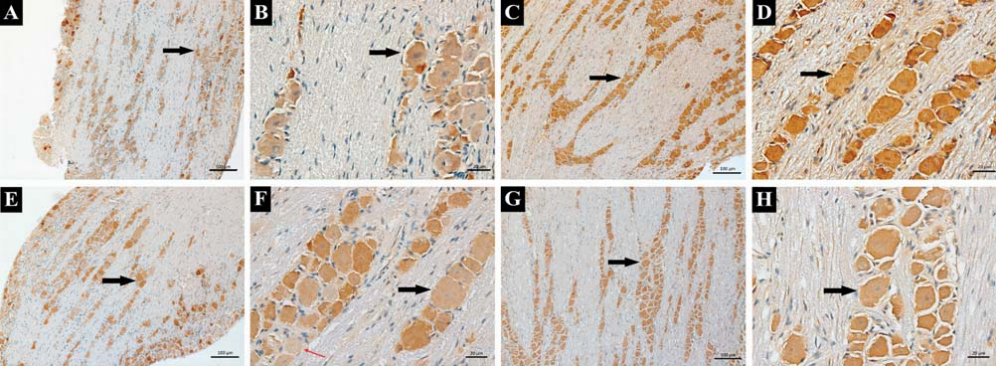
CGRP expressions in trigeminal ganglion (TG) following experimental tooth movement. A and B: CGRP were mainly expressed in TG neurons at baseline and stained in yellow-brown, as indicated by the black arrows (100x and 400x); C and D: CGRP was expressed in TG (as indicated by the black arrows) in the saline+force group at 3 d (100x and 400x); E and F: CGRP was expressed in TG (as indicated by the black arrows) in the small-dose capsaicin+force group at 3 d (100x and 400x); G and H: CGRP was expressed in TG (as indicated by the black arrows) in the large-dose capsaicin+force group at 3 d (100x and 400x). CGRP-negative cell is indicated by the red arrow

**Figure 3:**
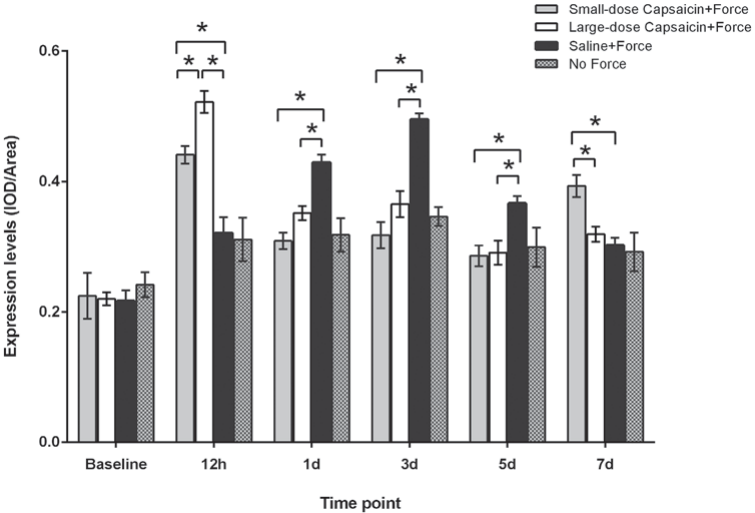
CGRP expression levels (IOD/Area) in trigeminal ganglion (TG) following experimental tooth movement investigated by immunohistochemical staining. * indicates p<0.05

As shown in Figure 2, CGRP was basically expressed in trigeminal neurons and CGRP-positive cells could also be observed at baseline (as indicated by the black arrow). As displayed in Figure 3, trends in CGRP expression levels (quantified through immunostaining intensity) for all the four groups were similar with those of western blotting. Specifically, for the small-dose capsaicin+force group, CGRP expression levels increased rapidly at 12 h, declined at 1 d, 3 d, and 5 d, and rebounded at 7 d. In contrast, for the large-dose capsaicin+force group, CGRP expression levels peaked at 12 h and decreased thereafter without rebounding at 7 d. Moreover, for the saline+force group, CGRP expression levels started to increase at 12 h, peaked at 3 d, and gradually decreased thereafter. In contrast, for the no force group, CGRP expression levels only increased at 3 d.

### The effects of capsaicin on CGRP expression in Vc following experimental tooth movement

**Figure 4:**
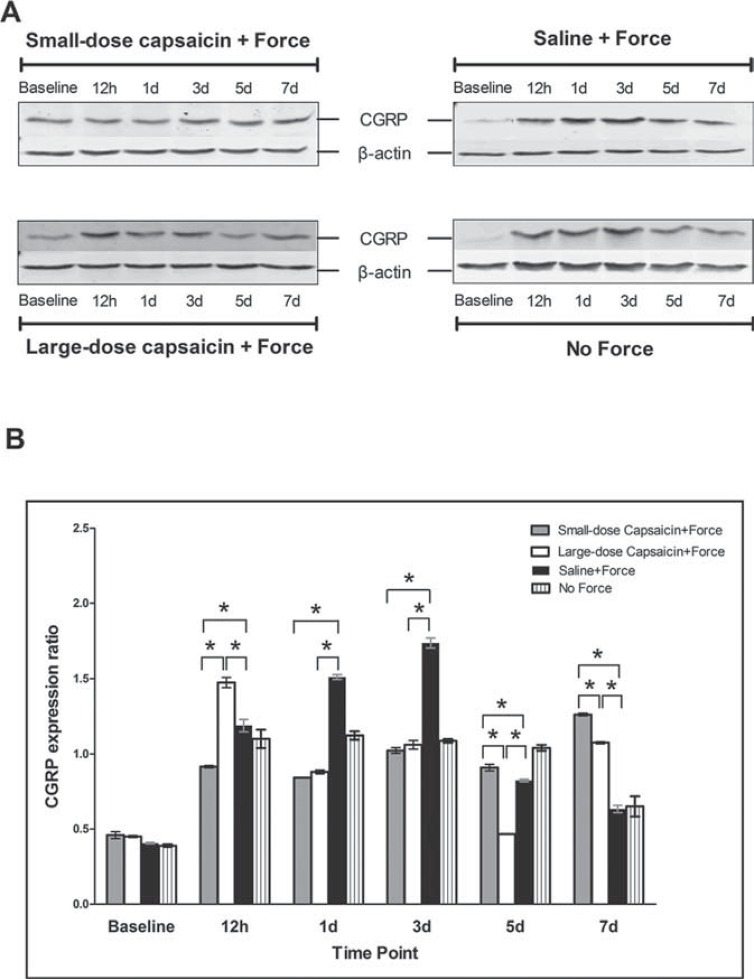
CGRP expressions in trigeminal subnucleus caudalis (Vc) following experimental tooth movement detected by western blotting. A: CGRP bands in Vc at different time points in the four groups; B: The histogram showed the expression ratio of CGRP protein levels normalized to that of β-actin in the same sample. * indicates p<0.05

We could also observe that bands of CGRP were shown near to 38 kDa, and that of β-actin near to 42 kDa (Figure 4A).

Compared with the baseline level (0.406±0.004), as displayed in Figure 4B, CGRP expression levels in the saline+force group increased at 12 h (1.187±0.041, p<0.001) and 1 d (1.511±0.017, p<0.001), peaked at 3 d (1.737±0.033, p<0.001), then gradually decreased at 5 d (0.823±0.008, p<0.001) and 7 d (0.633±0.024, p<0.001). Similarly, for the no force group, when compared with the baseline level (0.399±0.007), CGRP expression levels increased at 12 h (1.100±0.061, p<0.001), remained at the increased level at 1 d (1.122±0.029, p<0.001), 3 d (1.086±0.012, p<0.001), and 5 d (1.041±0.019, p<0.001), then decreased at 7 d (0.650±0.068, p<0.05).

On the other hand, CGRP expression levels were significantly higher in the large-dose capsaicin+force group than in the saline+force group at 12 h (1.474±0.034 vs. 1.187±0.041, p<0.05), then greatly decreased and became significantly lower than in the saline+force group at 1 d (0.880±0.012 vs. 1.511±0.017, p<0.0001), 3 d (1.061±0.028 vs. 1.737±0.033, p<0.0001), and 5 d (0.466±0.002 vs. 0.823±0.008, p<0.0001). Furthermore, CGRP expression levels in small-dose capsaicin+force group were significantly lower than in the saline+force group at 12 h (0.915±0.008 vs. 1.187±0.041, p<0.05), 1 d (0.842±0.003 vs. 1.511±0.017, p<0.0001), and 3 d (1.023±0.019 vs. 1.737±0.033, p<0.0001), but significantly higher than that in saline+force group at 5 d (0.909±0.021 vs. 0.823±0.008, p<0.05). In addition, CGRP expression levels both in the large-dose capsaicin+force and small-dose capsaicin+force groups gradually increased and became significantly higher than that in the saline+force group at 7 d (1.075±0.006 vs. 0.633±0.024, p<0.0001; 1.261±0.009 vs. 0.633±0.024, p<0.0001, respectively).

**Figure 5:**
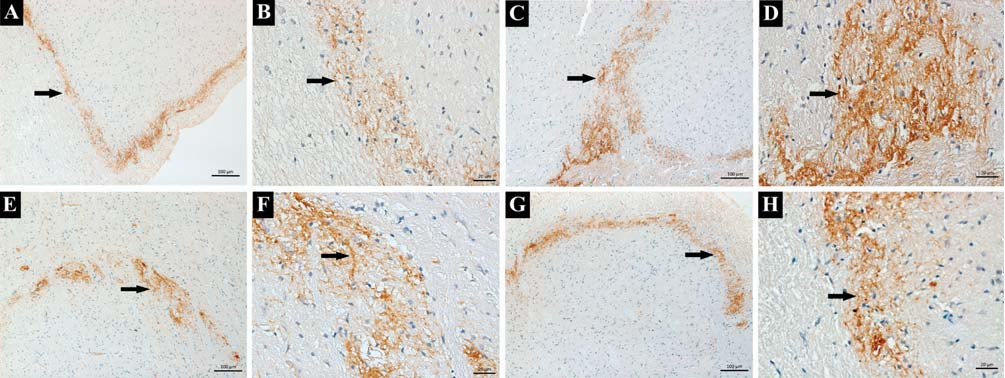
CGRP expressions in trigeminal subnucleus caudalis (Vc) following experimental tooth movement. CGRP-like immunoreactivity were mainly nerve bundles and displayed in network patterns stained yellow-brown, as indicated by the black arrows. A and B: CGRP expressions at baseline (100x and 400x). C and D: CGRP expression in the saline+force group at 3 d (100x and 400x); E and F: CGRP expression in the small-dose capsaicin+force group at 3 d (100x and 400x); G and H: CGRP expression in the large-dose capsaicin+force group at 3 d (100x and 400x)

**Figure 6:**
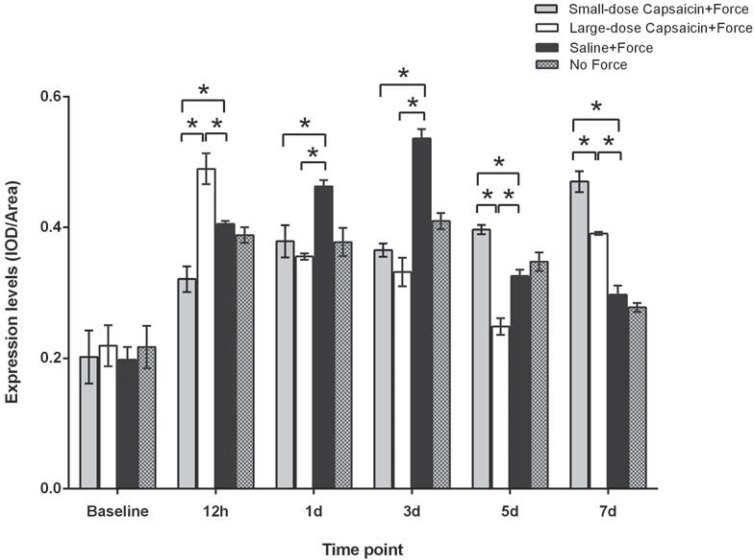
CGRP expression levels (IOD/Area) in trigeminal subnucleus caudalis (Vc) following experimental tooth movement investigated by immunohistochemical staining. * indicates p<0.05

As shown in Figure 5, CGRP-like immunoreactivity was observed in Vc at baseline, which displayed reticular formation and nerve fibers stained brown were the main structures. As presented in Figure 6, following experimental tooth movement, the chronological changes in CGRP expression levels were similar with those of western blotting. Specifically, for the small-dose capsaicin+force group, CGRP expression levels increased steadily from 12 h and peaked at 7 d. In contrast, for the large-dose capsaicin+force group, CGRP expression levels peaked at 12 h, declined thereafter until 5 d, and rebounded at 7 d. Moreover, for the saline+force group, CGRP expression levels began to increase at 12 h, peaked at 3 d, and dropped thereafter. For the no force group, CGRP expression levels increased and reached a plateau from 12 h to 5 d, and returned to baseline at 7 d.

## DISCUSSION

In this study, we found that capsaicin could regulate CGRP release both in TG and Vc following experimental tooth movement. Moreover, both small-dose and large-dose capsaicin could cause downregulation of CGRP expression at 1 d and 3 d, whereas a large CGRP release was observed in the saline+force group.

It is well-known that most patients could perceive orthodontic pain from 1 d to 3 d and pain sensation gradually subsides thereafter^22^, which has been the main adverse effect of orthodontic treatment. During experimental tooth movement, the force exerted on periodontal tissues could initiate local inflammation and cause the release of inflammatory mediators. Being the first-order sensory neurons in the orofacial region, TG could receive the nociceptive signal from peripheral nerve terminals located in periodontal tissues, and transmit it to the Vc. CGRP is exclusively synthesized in primary afferent neurons of the TG^12^. Previous studies have indicated that CGRP plays an important role in the underlying pathogenesis of pain conditions^4^. Our results showed that CGRP expression levels both in TG and Vc (the saline+force group) significantly increased at 1 d and peaked at 3 d, then gradually decreased at 5 d and 7 d. This change trend was consistent with a previous study, which indicated that CGRP was involved in the transmission and modulation of pain following experimental tooth movement^18^. Accordingly, we suggest that increased CGRP might reflect the effect of nociceptive stimuli on activation of trigeminal system and subsequent transmission and processing of this information in the brainstem^25^. However, ironically, CGRP expression levels increased in the no force group both in TG and Vc. Actually, since no force was exerted in this group, CGRP expression levels should be similar with those at baseline. We attribute it to discomforts and the buccal peculiar sense that might be caused by inactivated intraoral springs in rats^17^.

Our results showed that both small-dose capsaicin and large-dose capsaicin had an obvious impact on CGRP expression levels in TG and Vc following experimental tooth movement when compared with the saline+force group. It has been well-documented that capsaicin could selectively act on unmyelinated C-fibers and thin myelinated Aδ-fibers in primary sensory neurons by binding to TRPV1^6^. Activation of TRPV1 leads to an influx of calcium and CGRP release from peripheral nerve axons^13^ ^,^ ^29^. In our study, local periodontal injections of capsaicin caused a large CGRP release both in TG and Vc at 12 h following experimental tooth movement. The increased CGRP might partly reflect the stimulatory effect of capsaicin, which is known to activate sensory neurons and stimulate CGRP release^5^. It is worth mentioning that these two groups with different capsaicin dosages showed some differences in the CGRP expression levels at 12 h. The mechanism behind this effect might be the dose-dependent release of CGRP induced by excitation of TRPV1^24^. This could also be supported by the observation that application of capsaicin caused a dose-dependent release of salivary CGRP, which might reflect the activated state of trigeminal system^27^. Moreover, in our study, we found that following experimental tooth movement, CGRP expression levels both in TG and Vc at 1 d and 3 d were significantly lower than that in the saline+force group. One possible explanation for this phenomenon is based on the evidence that there is another effect of capsaicin, which is the inhibitory effect.^20^ that when recurrent application of capsaicin or administration of a single high dose, it could desensitize the voltage-gated calcium channels on sensory neurons^10^ and thereby could reduce the release of neurotransmitters. (This might be explained by that when recurrent or high dose of capsaicin is applied, it could desensitize the voltage-gated calcium channels on sensory neurons^10^. Subsequently, the release of neurotransmitters would be inhibited. Another explanation might be the large depletion of CGRP both in TG and Vc following injections of capsaicin and experimental tooth movement.

In addition, CGRP expression levels began to increase both in TG and Vc at 7 d after experimental tooth movement. We attribute this phenomenon to the beginning of CGRP synthesis in TG neurons and subsequent CGRP release in Vc. Regarding the difference of CGRP expression levels between small-dose+force group and large-dose+force group at the aforementioned time point, there are some evidence that might account for this. Previous studies have reported that after prolonged exposure to capsaicin or high-concentration capsaicin applied, it might initiate a process described as defunctionalization of voltage-gated calcium channels^1^, which is mainly due to the activation of calcium-dependent proteases^7^. This process could induce degeneration of capsaicin-sensitive nociceptive nerve endings^8^. We hereby hypothesized that large-dose capsaicin might cause a slower recovery of the TRPV1 activity, resulting in lower CGRP expression levels than small-dose capsaicin at 7 d.

**Figure 7:**
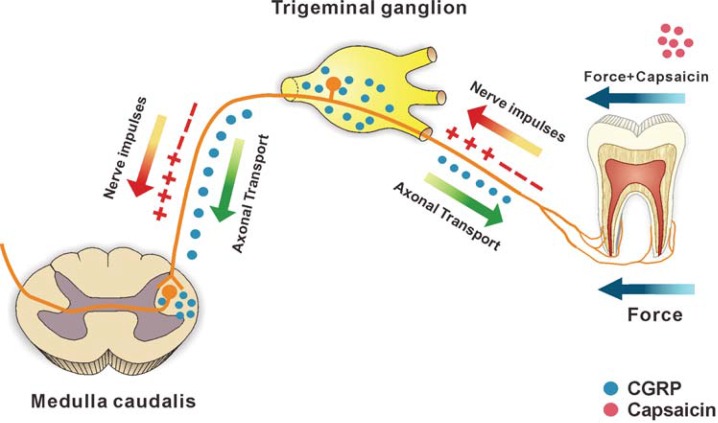
When orthodontic force was exerted on a tooth, nociceptive stimulus would be induced and perceived by peripheral nociceptors in periodontal tissues, where it could then generate pain signals that propagate to the TG. Once receiving these signals, TG would synthesize and release CGRP to periodontal tissues and Vc via bidirectional transport. After local injection of capsaicin, small-dose capsaicin could recruit more CGRP released from TG into periodontal tissues than Vc compared with large-dose capsaicin

Interestingly, in our study, we also found that the change trend of CGRP expression levels in Vc was not exactly the same as that in TG, especially in small-dose+force group. As previously mentioned, CGRP is mainly synthesized in TG and released both centrally and peripherally. Specifically, on one hand, peripheral released CGRP around blood vessels in periodontal ligament (PDL) originates from the TG^11^, which could also be supported by inferior alveolar nerve resection^14^. On the other hand, central processes of TG cells exhibiting CGRP-immunoreactivities almost completely terminate at the Vc^21^, which means that the released CGRP in Vc derives from the nerve extending from trigeminal neurons^26^. Based on this, we hypothesized that following experimental tooth movement, CGRP synthesized in TG might be bidirectionally transported to both periodontal tissues and Vc (Figure 7). Furthermore, we suggest that small-dose capsaicin was likely to stimulate CGRP release from TG more peripherally than centrally, thereby leading to the difference in CGRP expression levels between TG and Vc.

## CONCLUSION

Therefore, CGRP expression levels were elevated following experimental tooth movement both in TG and Vc, suggesting their involvement in the transmission of nociceptive information in tooth movement. Both small-dose and large-dose capsaicin could reduce CGRP expression levels both in TG and Vc following experimental tooth movement in rats at 1 d and 3 d. CGRP synthesized in TG neurons could be bidirectionally transported to peripheral tissues and Vc. Taken together, we suggest that capsaicin could regulate CGRP expression in TG and Vc following experimental tooth movement in rats. However, the underlying mechanism whereby capsaicin regulates CGRP expressions and the effect of capsaicin on orthodontic pain should be further elucidated.
